# Conditioned medium from amniotic mesenchymal tissue cells reduces progression of bleomycin-induced lung fibrosis

**DOI:** 10.3109/14653249.2011.613930

**Published:** 2011-09-28

**Authors:** Anna Cargnoni, Lorenzo Ressel, Daniele Rossi, Alessandro Poli, Davide Arienti, Guerino Lombardi, Ornella Parolini

**Affiliations:** 1 Centro di Ricerca E. Menni, Fondazione Poliambulanza-Istituto Ospedaliero, Brescia, Italy; 2 Dipartimento di Patologia Animale, Profilassi e Igiene degli Alimenti, Università di Pisa, Pisa, Italy; 3 Istituto Zooprofilattico Sperimentale della Lombardia e dell'Emilia Romagna, Reparto del Benessere Animale, Brescia, Italy

**Keywords:** amniotic cells, conditioned medium, human amniotic membrane, lung fibrosis, placenta-derived cells

## Abstract

**Background and aims:**

We have demonstrated recently that transplantation of placental membrane-derived cells reduces bleomycin-induced lung fibrosis in mice, despite a limited presence of transplanted cells in host lungs. Because placenta-derived cells are known to release factors with potential immunomodulatory and trophic activities, we hypothesized that transplanted cells may promote lung tissue repair via paracrine-acting molecules. To test this hypothesis, we examined whether administration of conditioned medium (CM) generated from human amniotic mesenchymal tissue cells (AMTC) was able to reduce lung fibrosis in this same animal model.

**Methods:**

Bleomycin-challenged mice were either treated with AMTC-CM or control medium, or were left untreated (Bleo group). After 9 and 14 days, the distribution and severity of lung fibrosis were assessed histologically with a scoring system. Collagen deposition was also evaluated by quantitative image analysis.

**Results:**

At day 14, lung fibrosis scores in AMTC-CM-treated mice were significantly lower (*P*<0.05) compared with mice of the Bleo group, in terms of fibrosis distribution [1.0 (interquartile range, IQR 0.9) versus 3.0 (IQR 1.8)], fibroblast proliferation [0.8 (IQR 0.4) versus 1.6 (IQR 1.0)], collagen deposition [1.4 (IQR 0.5) versus 2.0 (IQR 1.2)] and alveolar obliteration [2.3 (IQR 0.8) versus 3.2 (IQR 0.5)]. No differences were observed between mice of the Bleo group and mice treated with control medium. Quantitative analysis of collagen deposition confirmed these findings. Importantly, AMTC-CM treatment significantly reduced the fibrosis progression between the two observation time-points.

**Conclusions:**

This pilot study supports the notion that AMTC exert anti-fibrotic effects through release of yet unknown soluble factors.

## Introduction

Recent findings indicate compelling benefits of cell-based treatments in animal models of lung diseases associated with inflammatory and fibrotic processes (1). For example, transplantation of adult multipotent cells [e.g. bone marrow (BM)-derived mesenchymal stem cells (MSC) (2,3) and committed alveolar progenitor cells (4,5)], as well as transplantation of cells of fetal origin [such as placenta-derived cells (6,7) and human umbilical cord Wharton's jelly-derived cells (8)], has been shown to reduce lung fibrosis in animal models of bleomycin-induced pulmonary injury.

The mechanisms whereby these cell-based treatments are effective in reducing fibrosis in the lung remain poorly defined. Although initial interest focused on the level of engraftment of transplanted cells in host lungs and their potential for regenerating lung tissue by tissue-specific differentiation, the infrequency with which either occurrence has been documented makes it likely that additional mechanisms must account for the observed improvements (1). In this regard, we have reported previously that transplantation of either allogeneic or xenogeneic placental membrane-derived cells significantly reduced the severity of bleomycin-induced lung fibrosis in mice, despite a rare persistence of donor cells in the lungs of recipient animals (6). In addition, we found that human amniotic membrane patches reduced post-ischemic cardiac scars and liver fibrosis in rat models of coronary artery and bile duct ligation, respectively (9,10). Importantly, we did not observe detectable levels of amniotic membrane-derived cells in organs treated using these approaches. In other studies, we have shown that amniotic membrane-derived cells, and in particular cells isolated from the mesenchymal region of this membrane, exert immunomodulatory actions through the release of soluble factors (11,12). In addition, placental membrane-derived cells are able to secrete a number of biologically active molecules with potentially multifaceted activities, such as cytokines (13,14), angiogenic factors associated with wound healing (15) and growth factors related to cell proliferation and differentiation (15-17).

Based on these findings, we have put forward the hypothesis that the anti-fibrotic effects observed after transplantation of placental cells are mainly related to paracrine actions exerted on host tissues by bioactive molecules secreted by these cells. An important role for soluble factors in stem/progenitor cell-mediated reparative effects has been supported by animal experiments using conditioned culture medium (CM) as an effective treatment for different tissue injuries. Indeed, in a porcine model of myocardial ischemia and reper-fusion, intravenous and intracoronary injection of CM obtained from human MSC cultures reduced infarct size and improved cardiac performance (18). Furthermore, systemic infusion of human MSC-CM has been shown to reduce apoptosis and stimulate proliferation of hepatocytes in a rat model of acute liver injury (19). In addition, intramuscular injection of CM derived from human endothelial progenitor cells has resulted in tissue revascularization and functional recovery in a rat model of chronic hindlimb ischemia (20).

In this study, we evaluated the effects of administrating CM generated from cells derived from the mesenchymal region of human amniotic membrane (amniotic mesenchymal tissue, AMTC) (11) on bleomycin-induced lung fibrosis, and observed a reduction of fibrosis progression similar to that observed previously after allogeneic and xenogeneic placental membrane-derived cell transplantation. These proof of the principle results open the way to consideration of cell-free treatment approaches for fibrotic lung diseases.

## Methods

### AMTC isolation

Human term placentas (*n* = 8) were obtained following spontaneous vaginal delivery or Caesarean sections, with informed maternal consent according to the guidelines of the Institutional Ethics Committee (CEIOC, Ethics Committee of Catholic Hospital Institutions). After mechanical separation from the chorion, the amniotic membrane was washed extensively in phosphate-buffered saline (PBS; Sigma, St Louis, MO, USA) containing 100 U/mL penicillin and 100 μg/mL streptomycin (both from EuroClone, Wetherby, UK), and then cut into 3 × 3-cm fragments. Cells from the mesenchymal region of amniotic membrane were then isolated with a well established protocol that was set up at Centro di Ricerca E. Menni, Brescia, Italy, which is based on separation of the amniotic membrane from the chorionic membrane and subsequent enzymatic digestion, as described previously (11,21). We refer to these cells as AMTC (11,12). After passage (P) 2 in culture, these cells display a typical mesenchymal stromal phenotype, with the expression of markers such as CD90, CD73 and CD105>90% and the expression of hematopoietic markers, including CD45 and HLA-DR, < 2% (21,22). Freshly isolated AMTC display the following phenotype: CD90 (82 ± 3%),CD73 (66 ± 6%),CD 105 (6 ± 4%),CD44 (47 ±18%), CD166 (14 ±7%), CD45 (6 ± 3%) and HLA-DR (6 ± 3%) (similar to that previously reported by Magatti *et al*.) (11). Data are expressed as the mean of the percentage positive cells ± SD of at least 10 independent experiments.

### AMTC cultures and preparation of CM

Freshly isolated AMTC were plated in 24-well plates (Corning Inc., Corning, NY, USA) at a density of 1 × 10^6^ cells/well in serum-free culture medium (UltraCulture, Lonza, Basel, Switzerland) supplemented with 100 U/mL penicillin and 100 μg/mL streptomycin.

To generate AMTC-CM, cells were cultured for 5 days at 37°C in a humidified atmosphere of 5% CO_2_. Supernatants from each plate were then collected, pooled, centrifuged at *700 g*, filtered (0.2 μm) to remove cellular debris and stored at −80°C. This procedure was performed for cells obtained from eight different placentas. Finally, all of the collected supernatants were pooled, lyophilized and stored at 4°C until use, when they were dissolved in sterile water to one-quarter of the initial volume. Control, non-conditioned medium (non-CM) was generated in the same way as above, except that no cells were cultured in the plates.

### Experimental groups and CM injection

Animal experiments were carried out in accordance with current Italian and European regulations and laws on the use and care of animals for research (DL. 116/27 January 1992). All animal procedures were performed under general anesthesia (intraperitoneal injection of 2 mg/kg xylazine and 100 mg/kg ketamine).

Seventy 8–9-week-old C57BL/6 female mice (Charles River, Calco (Lecco), Italy), a strain reported to be bleomycin-sensitive (23), were instilled intratracheally with 4 U/kg bleomycin in a 50-μL volume, as described previously (6). Animals were then divided randomly into the following three experimental groups: Bleo group, mice that received no additional treatments; Bleo + non-CM group, mice receiving control non-CM treatment; Bleo + AMTC-CM group, mice receiving AMTC-CM treatment. As two mice died before they were to be euthanized (one belonging to the Bleo + non-CM group and one to the Bleo + AMTC-CM group), the three resulting groups analyzed at days 9 and 14 were as follows. Evaluation of fibrosis at day 9: Bleo group (*n* = 11), Bleo + non-CM group (*n* = 11) and Bleo + AMTC-CM group (*n* = 11). Evaluation of fibrosis at day 14: Bleo group (*n* = 12), Bleo + non-CM group (*n* = 11) and Bleo + AMTC-CM group (*n* = 12).

Fifteen minutes after bleomycin instillation, which was the same time-point at which we transplanted placental fetal membrane-derived cells in our previous study (6), 100 μL reconstituted medium was injected intrapulmonarily, in order to deliver the CM as close as possible to the site of damage. In detail, the anesthetized mice were placed in the left lateral decubitus and CM was injected percutaneously into the right fifth intercostal space using a 27-gauge needle. The needle was advanced approximately 8 mm into the thorax and quickly removed after injection. The quantity of medium injected corresponded with the volume of CM generated by 4 × 10^5^ cultured cells, a number comparable with the number of placental mesenchymal cells that were transplanted into each mouse in previous experiments (6).

### Lung fibrosis evaluation

Animals of each experimental group were euthanized at day 9 or day 14 after bleomycin instillation. Whole lungs of each mouse were formalin-fixed for 48 h and embedded in paraffin. Each block was cut at two different coronal section planes (at approximately 500 μm distance from each other), representative of two regions of the maximum observable lung area. Consecutive 4-μm thick sections were cut and mounted on Superfrost slides (Thermo Scientific, Menzel GmbH & Co. KG, Braunshweig, Germany) and dried overnight at 37°C. For each lung, two consecutive sections for each plane were stained with hematoxylin-eosin (HE) and Masson-Goldner trichrome (MGT).

Histologic grading of fibrosis was performed by a veterinary pathologist, who was blinded to the experimental conditions, using a bright-field microscope and following a semi-quantitative method adapted from Hagood *et al*. (24). The fibrotic process was assessed by scoring four parameters: fibrosis distribution, collagen deposition (both on MGT-stained sections), fibroblast proliferation and alveolar obliteration (both on HE-stained sections). Fibrosis distribution evaluates semi-quantitatively the amount of lung area across the whole section that is affected by the fibrotic process, and is expressed as 1, 2, 3 or 4, when 1–25%, 26–50%, 51–75% or 76–100% of the lung area is fibrotic, respectively. Severity parameters (fibroblast proliferation, collagen deposition and alveolar obliteration) were scored as 1, 2 or 3 when pathologic alterations were graded as mild, moderate or severe, respectively. Specifically, fibroblast proliferation evaluates the presence of spindle cells, morphologically indicative of fibroblasts. Collagen deposition evaluates the presence of green-stained collagen areas, and alveolar obliteration evaluates the amount of area characterized by the thickening of interalveolar septa that reduces alveolar spaces. For each animal, the fibrosis distribution score and the fibrosis severity parameter mean score (of six fibrosis-representative, randomly chosen, non-overlapping high power fields for each section) were represented by the average of the scores obtained from the two examined sections. The overall fibrosis score for each animal was obtained by taking the sum of each mean score of the fibrosis severity parameters and multiplying this result by the fibrosis distribution mean score.

### Collagen deposition image morphometric analysis

To evaluate collagen deposition quantitatively, we adopted a previously described image analysis-based system (10). Briefly, images of MGT-stained specimens were captured with a digital camera (Olympus Camedia C-4040 ZOOM) and digitalized at 1024 × 768 pixel, 24-bit/pixel resolution with a global magnification of × 400. Digital images were processed to identify, isolate and measure areas occupied by green-stained collagen deposition as a percentage of the total lung parenchyma area included in each field. The mean value of measurements obtained from three non-overlapping fields per section showing fibrotic processes was assigned to each animal.

### Statistical analysis

Data are expressed as median values and the relative interquartile range (IQR). In the figures, the data are represented by box-plot, where the band inside the box represents the median value, the box represents the IQR and whiskers show the distribution of the data. Comparisons between values collected at day 9 with those collected at day 14 for the same experimental group was performed by using the Mann-Whitney test (Table I). Comparisons between more than two groups (in order to compare results obtained at each time-point for the three experimental groups) were performed by using the Kruskal-Wallis non-parametric analysis of variance. In cases of statistical significance, the Mann-Whitney test using Holm-Bonferroni's correction for multiple comparisons was applied to assess the differences between experimental groups at the specific time-points (at day 9 or at day 14). A *P*-value<0.05 was considered statistically significant. Statistical analysis was performed with SPSS Advanced Statistics 13.0 (SPSS Inc., Chicago, IL, USA).

**Table I:** Comparison of fibrosis parameters between days 9 and 14 within each experimental group.

	Bleo group					Bleo + non-CM group					Bleo + AMTC-CM group				
			
	Day 9		Day 14			Day 9		Day 14			Day 9		Day 14		
									
	M	IQR	M	IQR	P	M	IQR	M	IQR	P	M	IQR	M	IQR	P
Fibrosis distribution	1.5	1.0	3.0	1.8	< 0.05	1.0	0.5	2.5	2.0	< 0.01	1.0	0.3	1.0	0.9	NS
Fibroblast proliferation	0.7	0.4	1.6	1.0	< 0.01	0.2	0.3	1.5	1.2	< 0.01	0.7	0.5	0.8	0.4	NS
Collagen deposition	1.2	0.3	2.0	1.2	< 0.05	1.0	0.8	2.2	1.0	< 0.01	1.0	0.5	1.4	0.5	NS
Alveolar obliteration	2.7	0.8	3.2	0.5	NS	2.3	0.8	3.1	1.2	< 0.05	2.5	1.2	2.3	0.8	NS

Fibrosis progression was observed in the control groups (Bleo and Bleo + non-CM) from days 9 to 14, but was absent in the AMTC-CM treated group.

Median (M) with IQR of scores obtained from animals of the same group at each time-point. The statistical differences between time-points of each group are reported as *P*-values, which were calculated as described in the Methods. NS, not significant.

## Results and discussion

**Figure 1:**
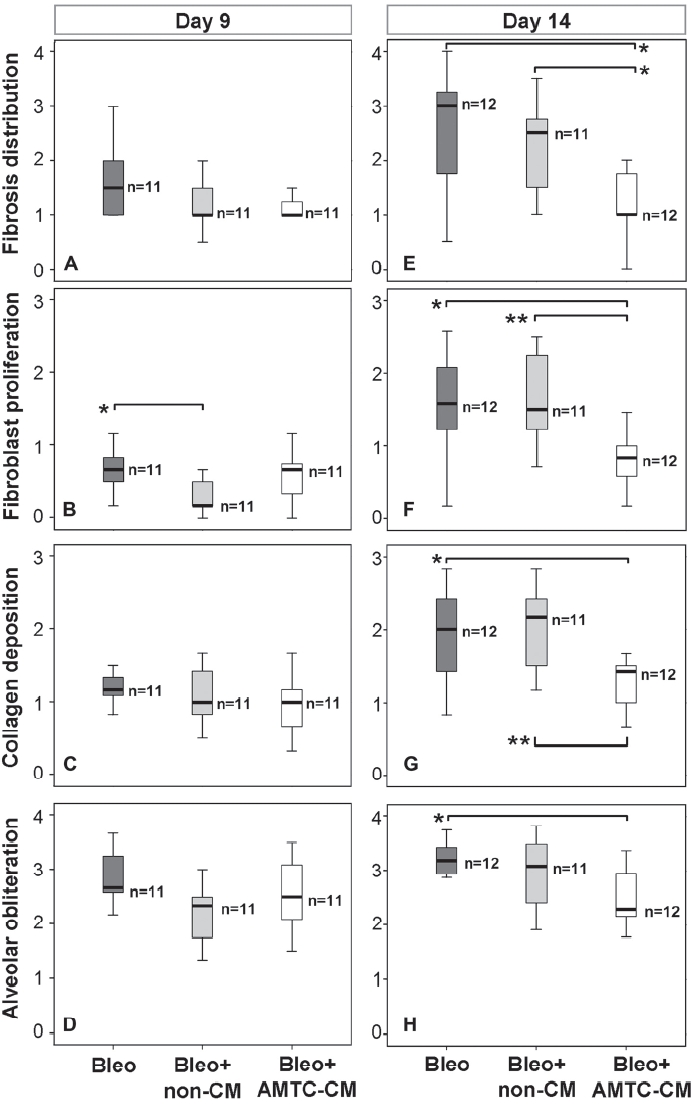
Representation of fibrosis distribution and severity parameter scores at days 9 and 14 post-intratracheal bleomycin instillation. (A-D) Fibrosis distribution (A) and severity (B-D) at day 9. (E-H) Fibrosis distribution (E) and severity (F-H) at day 14. (A, E) Fibrosis distribution; (B, F) fibroblast proliferation; (C, G) collagen deposition; (D, H) alveolar obliteration. The number of mice in each group is indicated (*n*). Brackets represent significant differences between groups; *P<0.05; **P<0.01.

The aim of this study was to investigate the potential beneficial effects of AMTC-CM on bleomycin-induced lung fibrosis. We analyzed the effects of AMTC-CM administration on fibrosis distribution, a parameter that reveals the amount of lung area affected by the fibrotic process across the entire section examined. At day 9, about one-third of the lung area was affected by the fibrotic process in all three experimental groups, with no differences observed among the groups (Figure 1A). At day 14, in mice left untreated and those treated with non-CM, the fibrotic process had developed further and occupied about two-thirds of the total area examined. The scores for fibrosis distribution in these two groups did not differ (Figure 1E). In contrast, samples obtained at this time-point from the group of mice treated with AMTC-CM showed that only one-third of the lung area remained affected by the fibrotic process, which resulted in a fibrotic score that was significantly reduced compared with the two control groups, with a median score of 1.00 (IQR 0.9) versus 3.0 (IQR 1.8) for the Bleo group (*P*<0.05) and 2.5 (IQR 2.0) for the Bleo + non-CM group (*P*<0.05) (Figure 1E).

To investigate further the potential anti-fibrotic actions of AMTC-CM, each lung area that had previously been identified as fibrotic was analyzed and scored for fibroblast proliferation, collagen deposition and alveolar obliteration, the three main pathologic hallmarks used to describe the severity of bleomycin-induced fibrotic alterations (24). At day 9, the median scores for each of the three parameters were *not significantly* different among the three experimental groups (Figure 1B-D), with the exception of the score for fibroblast proliferation, which was lower in the samples from mice treated with non-CM compared with mice left untreated (Figure 1B). This difference was no longer evident at day 14 (Figure 1F). However, at day 14, samples from mice of the control groups showed worsening fibrosis, with more severe fibroblast proliferation, increased collagen accumulation and alveolar obliteration (Figure 1F-H). In contrast, in samples from mice treated with AMTC-CM, the scores for all of these parameters remained similar to those observed at day 9 and were significantly lower than those observed in both control groups, with the exception of the alveolar obliteration parameter, where the difference between the samples obtained from AMTC-CM-treated animals and the non-CM-treated controls did not reach statistical significance (Figure 1F-H). Specifically, when compared with the other two groups, AMTC-CM-treated mice showed lower fibroblast proliferation [0.8 (IQR 0.4) versus 1.6 (IQR 1.0) for the Bleo group (*P*<0.05), and versus 1.5 (IQR 1.2) for the Bleo + non-CM group (*P*< 0.01); Figure 1F], collagen deposition [1.4 (IQR 0.5) versus 2.00 (IQR 1.2) for the Bleo group (*P*<0.05), and versus 2.2 (IQR 1.0) for the Bleo + non-CM group (*P*<0.01); Figure 1G] and alveolar obliteration [2.3 (IQR 0.8) versus 3.2 (IQR 0.5) for the Bleo group (*P*<0.05), and versus 3.1 (IQR 1.2) for the Bleo + non-CM group; Figure 1H]. No differences were observed among scores for any of the parameters measured in lung samples from mice that were left untreated or were treated with non-CM (Figure 1F-H).

**Figure 2:**
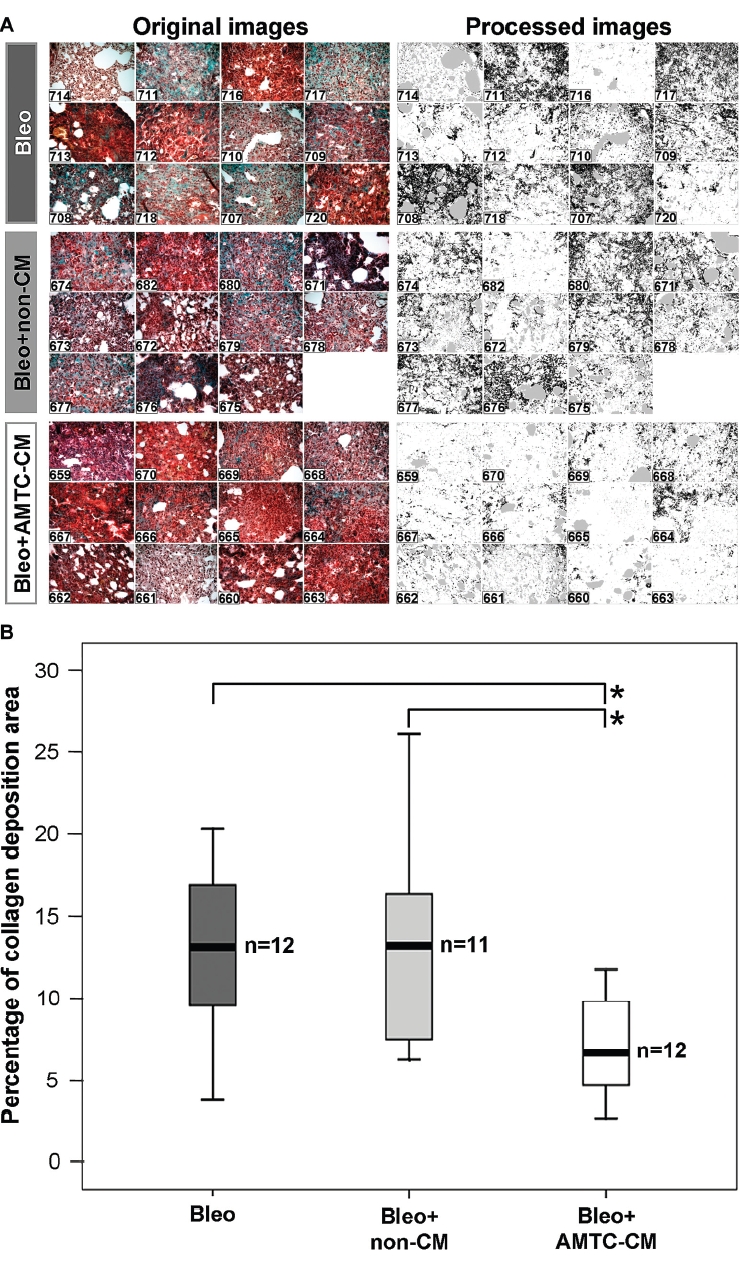
Digital image morphometric analysis of lung collagen deposition. (A) Microphotographs taken before (left panel) and after (right panel) the imaging analysis procedure used to quantitate the collagen staining (black areas) from the image. The identification number of each mouse is indicated on each microphotograph. (B) Median values with IQR of quantitative collagen deposition are represented as box-plots for Bleo (dark gray), Bleo + non-CM (light gray) and Bleo + AMTC-CM (white) groups. The number of mice in each group is indicated (*n*). Brackets represent significant differences between groups; *P<0.05.

Quantitative image morphometric analysis (10) of fibrotic areas confirmed the above findings and showed a significant reduction in collagen deposition at day 14 in samples from the mice treated with AMTC-CM compared with the two control groups, with a median value of 6.6% (IQR 5.5) versus 13.2% (IQR 7.5) for the Bleo group (*P*<0.05) and 13.2% (IQR 9.6) for the Bleo + non-CM group (*P*<0.05) (Figure 2A, B).

Comparison of scores for each lung fibrosis parameter at the two observation time-points revealed significant worsening of all parameters in samples from mice from the two control groups, with the single exception of alveolar obliteration, which did not increase significantly from day 9 to day 14 in the mice that were left untreated. Conversely, samples from lungs of mice treated with AMTC-CM showed no significant increase in any of the four measured lung fibrosis parameters (Table I).

**Figure 3:**
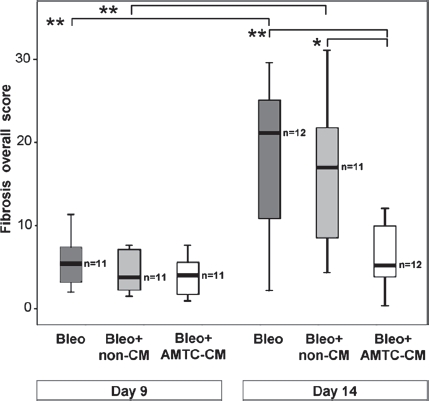
Representation of overall fibrosis score at days 9 and 14 post-intratracheal bleomycin instillation. Box-plots reporting the median and IQR of values obtained from the Bleo (dark gray), Bleo + non-CM (light gray) and Bleo + AMTC-CM (white) groups are represented for each time-point. The number of mice in each group is indicated (*n*). Brackets represent significant differences between groups and time-points; *P<0.05; **P<0.01.

These results were also confirmed with the analysis of the overall fibrosis score, which indicated significant reduction in severity and prevention of progression of lung fibrosis in mice treated with AMTC-CM 14 days after bleomycin instillation. These mice showed a score of 4.9 (IQR 6.6) compared to a score of 20.8 (IQR 15.4), (*P*<0.01) and 16.7 (IQR 19.3) (*P*< 0.05) observed in untreated and non-CM-treated control mice, respectively (Figure 3).

The experimental design used in our studies was based on data collection 9 and 14 days after intratracheal bleomycin instillation. The use of these time-points avoids the confounding aspects of the early inflammatory response and allows scoring of the lung fibrotic process at its intermediate and maximum levels, as we and others have demonstrated previously (2,3,6,25-27). Indeed, studies performed 21 and 28 days after bleomycin instillation do not show significant differences in the fibrotic process compared with that described at day 14 (A. Cargnoni *et al*., unpublished observations).

In addition to the same exhaustive histologic assessment of fibrosis on the whole lung tissue (without subsampling the lungs) of experimental animals that we have applied in previous studies (6), the analysis of our current experiments was enhanced by the addition of a quantitative evaluation of collagen deposition performed by a digital image morphometric analysis (28). Our findings show that the administration of AMTC-CM prevents the progression of bleomycin-induced lung fibrosis and results in a significant reduction in the parameters related to the fibrotic process at day 14 after intra-tracheal bleomycin instillation. These results are consistent with the beneficial effects of transplanted fetal membrane-derived cells that we have observed previously in the same animal model (6), and provides proof of the principle that AMTC-CM can ameliorate the outcome of acute toxic insult of the lung if administered soon after induction of the injury.

It is noteworthy that, even though AMTC-CM was injected into the right lung, a reduction in pulmonary fibrosis was observed in both the left and right lung fields, with no detectable differences between the two lungs in each mouse. It is conceivable that AMTC-CM injected into the right lung can reach the left lung through the exchange of fluid that occurs between communicating airways during respiratory function or, possibly, via the systemic circulation after absorption by alveolar capillaries, as we have detected donor cells in the left lungs of mice after injection of amniotic membrane-derived cells in the right lung (Cargnoni et al., unpublished observations).

Because virtually no effects on the fibrotic process were observed in mice receiving administration of non-CM, we conclude that AMTC-CM contained soluble molecules released by AMTC that were responsible for the reduction in severity and progression of the fibrotic reaction. Considering that increasing evidence has highlighted recently that MSC isolated from various sources produce bioactive molecules, which are potentially able to exert several types of paracrine effects (e.g. anti-scarring, anti-inflammatory, anti-apoptotic) on target cells (29), and that injection of CM obtained from MSC is an effective experimental treatment for different tissue injuries (e.g. 18,19), interesting results will no doubt be obtained from comparative studies in the same animal model that will investigate whether CM generated from cells derived from sources other than the amniotic membrane can also reduce lung fibrosis in a manner similar to AMTC-CM.

Determination of the nature and characteristics of the paracrine soluble molecules involved in AMTC-CM-mediated fibrosis reduction, as well as their mechanism(s) of action and their target cells, is obviously a task of major importance that warrants further extensive investigation and is the object of ongoing studies. Similarly, trying to understand the mechanisms underlying the beneficial effects of paracrine actions performed by stem cells in general remains a challenge for all researchers in the field.

It is tempting to speculate that a cell-free treatment, based on the use of AMTC-CM, could potentially replace cell transplantation, particularly when tissue repair via paracrine bioactive molecules rather than tissue regeneration via cell replacement/differentiation is required, with this strategy also offering a series of added advantages. In particular, AMTC-CM can be produced easily and in large quantities; it can be stored efficiently because it maintains its anti-fibrotic efficacy after the lyophilization process; as a cell-free treatment, it can drastically reduce the risk of adverse immuno-logic reactions, infectious risks and other potential long-term negative effects caused by the presence of exogenous cells; finally, it is also conceivable that AMTC-CM could be administered safely via intravenous injection, avoiding clot formation and lung capillary entrapment.

In conclusion, the present study contributes to the body of knowledge regarding the properties of amniotic membrane-derived cells (and specifically of AMTC) and envisages a new potential use for the multifaceted immunomodulatory and trophic features of these cells through the application of cell-free preparations obtained from culture medium generated by these cells. Despite the fact that this study does not contribute to the identification of which paracrine factor(s) produced by AMTC and released into the CM could be involved in the observed reduction in fibrosis, and also the fact that further studies are required to understand the paracrine mechanisms involved, as well as to investigate the effects of AMTC-CM on already established fibrosis and compare the effects of CM produced by other cell types, our results point to AMTC-CM as a potential new tool that deserves consideration in the development of novel strategies and clinical applications for lung fibrosis.
